# Prevalence and factors associated with suicidal ideation among students taking university entrance tests: revisited and a study based on Geographic Information System data

**DOI:** 10.1192/bjo.2023.526

**Published:** 2023-07-18

**Authors:** Rifat Nahrin, Firoj Al-Mamun, Mark Mohan Kaggwa, Md. Al Mamun, Mohammed A. Mamun

**Affiliations:** Department of Economics, Comilla University, Cumilla, Bangladesh; CHINTA Research Bangladesh, Savar, Dhaka, Bangladesh; Department of Public Health and Informatics, Jahangirnagar University, Savar, Dhaka, Bangladesh; and Department of Public Health, University of South Asia, Dhaka, Bangladesh; Department of Psychiatry, Faculty of Medicine, Mbarara University of Science and Technology, Mbarara, Uganda; and Department of Psychiatry and Behavioral Neurosciences, McMaster University, Hamilton, Ontario, Canada; Department of Public Health and Informatics, Jahangirnagar University, Savar, Dhaka, Bangladesh; CHINTA Research Bangladesh, Savar, Dhaka, Bangladesh; and Department of Public Health and Informatics, Jahangirnagar University, Savar, Dhaka, Bangladesh

**Keywords:** Suicidal behaviour, suicide, depression, suicidal ideation, university student

## Abstract

**Background:**

A previous study identified students taking Bangladeshi university entrance tests as a vulnerable group at a higher risk of suicidal behaviours before the COVID-19 pandemic. However, the impact of the pandemic on the magnitude of these behaviours among this population remains unexplored.

**Aims:**

This study aimed to investigate the prevalence of suicidal ideation and associated factors among Bangladeshi university entrance test takers following the pandemic. In addition, an approach based on Geographic Information System (GIS) data was used to visualise the distribution of suicidal ideation across the country.

**Methods:**

A cross-sectional approach was used to collect data among participants taking the entrance test at Jahangirnagar University in September 2022. Using SPSS, data were analysed with chi-squared tests and binary regression, and ArcGIS was used to map the results across the nation.

**Results:**

The study revealed a prevalence of 14.4% for past-year suicidal ideation, with 7.4% and 7.2% reporting suicide plans and attempts, respectively. Notably, repeat test-takers exhibited a higher prevalence of suicidal behaviours. Significant risk factors for suicidal ideation included urban residence, smoking, drug use, COVID-19 infection and deaths among close relations, depression, anxiety and burnout. The GIS-based distribution indicated significant variation in the prevalence of suicidal ideation across different districts, with higher rates observed in economically and infrastructurally deprived areas.

**Conclusions:**

Urgent measures are needed to address the high prevalence of suicidal behaviours among students taking university entrance tests students in Bangladesh, particularly in light of the COVID-19 pandemic. Enhanced mental health support, targeted prevention efforts and improved resources in economically disadvantaged regions are crucial to safeguard the well-being of these students.

Evidence suggests a growing trend of mental health problems among students, particularly high-school and university students.^1^ When high-school students transition to university, they undergo various educational, social and emotional changes.^2^ The period of transitioning from high school to university can be highly stressful for these students. In many countries, including Bangladesh, India, Germany, Russia and France, high-school graduates must pass an entrance exam to gain admission to tertiary education.^3^ Obtaining a university degree is considered to be a significant accomplishment, as it equips individuals with new patterns and skills, immersing them in a vast ocean of knowledge.^4^

In 2019, of the 1 336 629 Bangladeshi students who took the high-school graduation exam, 74% successfully passed, making most of them eligible for the university entrance test.^5^ However, in 2020, the exam was cancelled because of the COVID-19 pandemic, resulting in a 100% pass rate based on previous public examination results. In 2022, with a curriculum focused on only three subjects, the pass rate stood at 95.26%, but it dropped to 85.96% when students were required to take 12 tests covering six subjects the following year.^6^ Despite this, many students still qualify for the university admission test, although the capacity of higher education in public institutes of the country is limited to around 55 000.^7^ The consequent competitive nature of this exam, along with associated activities such as preparing for different exam syllabuses for various institutes and/or faculties (medical colleges, engineering faculties, agricultural universities, etc.) and presenting for entrance tests at different locations, can substantially affect the mental health of high-school graduates. Reports indicate that many Bangladeshi students presenting for university entrance tests experience mental health problems, with approximately 47.9 and 28.9% reporting depression and anxiety, respectively, and 43.7% exhibiting symptoms of burnout.^3,8^ Considering the strong association between mental health problems and suicidality,^4,9^ it is unsurprising that students taking university entrance tests are at a higher risk of suicidal behaviour. In fact, 17.7% of these students reported suicidal ideation in a previous study, and 8.0 and 2.5% reported having made suicide plans and attempts, respectively.^3^

It is crucial to emphasise that suicide is not a solution to any stressful situation. Unfortunately, there has been an increase in suicidal behaviours among Bangladeshi individuals, particularly after the onset of the COVID-19 pandemic, as described by Mamun's review.^10^ As mentioned earlier, the traditional schooling and graduation system underwent significant changes during the pandemic, with students being auto-passed without taking any exams or taking the test with a shortened syllabus. Consequently, many students achieved higher grade point averages (GPAs).^6^ However, those who had worked hard to attain the highest GPA results may have experienced emotional distress and a loss of confidence when preparing for the entrance test, as the GPA contributes to the overall entrance test scores. Moreover, pandemic-related challenges such as contracting COVID-19, witnessing loved ones being infected, and experiencing the loss of someone close owing to the virus could have profoundly affected the psychological well-being of students preparing for the entrance test.^11^

It is probable that rates of suicidal behaviours and factors associated with suicidal ideation among students taking university entrance tests may have changed (i.e. increased) since the onset of the pandemic; however no relevant study has been conducted after the pandemic's inception. Thus, the present study investigates the prevalence of suicidal behaviours among Bangladeshi students presenting for university entrance tests and examines the factors associated with suicidal ideation, comparing the results with those of the previous study by Mamun et al.^3^ This study also presents a nationwide distribution of suicidal ideation using Geographic Information System (GIS) data, based on the districts where participants reside, including gender-based and student-status-based distributions, to visualise the severity of suicidal ideation in different districts and facilitate targeted suicide-prevention efforts in high-risk regions.

## Method

### Study participants and procedure

This cross-sectional study was conducted among students taking the university entrance test at Jahangirnagar University, Dhaka, Bangladesh. The entrance test was held between 4 and 11 September 2022, and the data were collected within this period. Test-taking students who resided in university dorms at the time of the entrance test were eligible for this study. A few rooms in each dorm were allocated for residing students, making the data collection approach easy. A convenience sampling approach was followed in this study, in which every test-taking student present in the relevant dorms at the time of the survey was approached and participated in the study; that is, the response rate was 100% A self-reporting survey was conducted to collect the responses, before which the participants were briefed about the terms used in the survey questionnaire. Initially, a total of 1574 responses were collected; after removal of incomplete questionnaires, data from 1523 participants were analysed.

### Measures

#### Sociodemographic factors

Sociodemographic information including gender, permanent residence (city versus village), monthly family income, religion, smoking status and substance use status was collected in the survey. As in the previous study,^3^ monthly family income was grouped into three categories: less than 15 000 Bangladeshi Taka (BDT), 15 000–30 000 BDT, and more than 30 000 BDT.

#### COVID-19-related factors

A total of three questions related to COVID-19 were asked of the participants. First, they were asked whether they had been infected with COVID-19. Later, information related to any family members or friends of participants who had been infected with COVID-19 or died owing to COVID-19 was also collected based on binary (yes/no) responses.

#### Admission-related variables

In Bangladesh, most universities allow students to take the entrance test twice. Information about participants’ test-taking status, i.e. whether they were first-time or repeat test takers, was collected. Information related to previous public examination tests at high schools, such as educational background or dimension and GPA, was also collected. Students were further asked about mock test performance and whether they had been guided by any professional or coaching centre when preparing for the test. In addition, the survey asked for information including test-related monthly expenditures and the type of university to which the participant was seeking admission.

#### Patient Health Questionnaire

The Patient Health Questionnaire (PHQ-9) was used to assess depression in this study.^12^ The PHQ-9 comprises a total of nine items that are responded to using a four-point Likert scale (0 = not at all, 1 = several days, 2 = more than half of the days and 3 = nearly every day) based on the past 2 weeks. The scale ranges from 0 to 27, where a higher score indicates greater depression severity. A score equal to or greater than 10 is considered to indicate depression.^12,13^ In the present study, Cronbach's alpha was 0.76.

#### Generalized Anxiety Disorder questionnaire

The Generalized Anxiety Disorder (GAD-7) questionnaire was used to assess anxiety in this study.^14^ The GAD-7 comprises a total of seven items that are responded to using a four-point Likert scale (0 = not at all, 1 = several days, 2 = more than half of the days and 3 = nearly every day) based on the past 2 weeks. The scale ranges from 0 to 21, where a higher score indicates greater anxiety severity. A score equal to or greater than 10 is considered to indicate anxiety.^13,14^ In the present study, Cronbach's alpha was good (0.83).

#### Maslach Burnout Inventory – Student Survey

The Maslach Burnout Inventory – Student Survey (MBI-SS) was used to assess burnout among students in this study.^15^ The MBI-SS comprises 15 items scored on a seven-point Likert scale ranging from 0 (strongly disagree) to 6 (strongly agree). It contains three subscales: exhaustion (four items), cynicism (five items) and efficacy (six items). The scoring of the subscales is as follows: emotional exhaustion (low = 0–9, moderate = 10–14, high >14); cynicism (low = 0–1, moderate = 2–6, high >6); and academic efficacy (low ≤22, moderate = 23–27, high ≥28).^16^ To determine burnout, a two-dimensional approach was used, whereby participants were classified as having burnout if they scored ‘high’ for both emotional exhaustion and cynicism.^17^ In the present study, Cronbach's alpha was good (0.8).

#### Suicidal behaviours

In this study, three types of suicidal behaviours were evaluated: suicidal ideation, suicide planning and suicide attempts. In a single question, participants were asked whether they had experienced thoughts of dying by suicide, indicating suicidal ideation. In addition, they were queried about any plans or attempts to die by suicide. The assessment of these behaviours followed a similar approach to that used by a previous study conducted among students taking university entrance tests before the COVID-19 pandemic, using binary response options (yes/no).^3,10,18^ The timeframe for assessing all three types of suicidal behaviours was the past year.

### Statistical analysis

Data collection and entry were performed using Google forms, and data were then formatted as an SPSS file for the final analysis. Descriptive and inferential statistics were used to analyse the data, and all the analyses were performed for the total sample and for subgroups based on student status (i.e., first-time versus repeat test takers). For descriptive statistics, frequencies and percentages were used, and chi-squared test and binary logistic regression were used to determine associations between the studied variables and suicidal ideation. A *P*-value of 0.05 was considered to indicate significance in all tests, and a 95% confidence interval was taken as standard in this study. In addition, ArcGIS 10.8 software was used for spatial analysis of suicidal ideation, with geographic data downloaded from https://www.diva-gis.org/. First, total participant data were obtained by district; then, two *post hoc* analyses were performed, and the results are presented in map form based on gender and student status. Maps from government mapping sites, which are free to use, were collected for presentation of the results.

### Ethical considerations

The authors assert that all procedures contributing to this work comply with the ethical standards of the relevant national and institutional committees on human experimentation and with the Helsinki Declaration of 1975, as revised in 2008. All procedures involving human subjects and/or patients were approved by the review board of CHINTA Research Bangladesh. Before participants were invited to enrol in this study, they were briefed about the study and its aims and objectives, and about their right to refuse to participate or withdraw at any time.

### Consent statement

Each participant signed a written consent form before starting the survey.

## Results

### Description of the study participants

A total of 1523 participants enrolled in the study; most of them were male (76.8%, *n* = 1169), rural residents and Muslim in religion and belonged to a nuclear family. About 10.3% of the test-taking students reported smoking, whereas only 4% reported drug use. Regarding the COVID-19-related information, about 8.4% reported having been infected with the virus, and 18.3 and 9.6% had experienced someone with whom they had a close relationship being infected with the virus or dying because of it, respectively. Regarding student status, 71.5% were first-time test takers and the remainder were repeat test takers. About 73.1% received professional help when preparing for the test, and 37.5% reported being satisfied with their performance on mock tests. More than half of the participants were depressed (53.8%), one-third were anxious (33.2%) and 34.4% had burnout (Table 1).

**Table 1:** Distribution of variables by student status (first-time versus repeat test taker)

Variable	Total, *n* (%)	First-time test taker, *n* (%)	Repeat test taker, *n* (%)
**Sociodemographic variables**			
Gender			
Male	1169 (76.8)	826 (75.8)	343 (79)
Female	354 (23.2)	263 (24.2)	91 (21)
Permanent residence			
Rural	1127 (74.9)	824 (76.7)	303 (70.5)
Urban	377 (25.1)	250 (23.3)	127 (29.5)
Religion			
Muslim	1298 (87.1)	937 (88)	361 (84.9)
Other	192 (12.9)	128 (12)	64 (15.1)
Family type			
Nuclear	1115 (76.2)	808 (77.5)	307 (72.9)
Joint	348 (23.8)	234 (22.5)	114 (27.1)
Monthly income (BDT)			
<15 000	375 (36.5)	266 (37.8)	109 (33.5)
15 000–3000	383 (37.3)	263 (37.4)	120 (36.9)
>30 000	270 (26.3)	174 (24.8)	96 (29.5)
Cigarette smoking			
Yes	152 (10.3)	97 (9.2)	55 (12.9)
No	1328 (89.7)	955 (90.8)	373 (87.1)
Drug usage			
Yes	59 (4)	40 (3.8)	19 (4.4)
No	1422 (96)	1017 (96.2)	405 (93.3)
**COVID-19 related information**			
Personal COVID-19 infection			
Yes	126 (8.4)	88 (8.2)	38 (8.9)
No	1373 (91.6)	985 (91.8)	388 (91.1)
Family/friend's COVID-19 infection			
Yes	275 (18.3)	193 (18)	82 (19.2)
No	1224 (81.7)	880 (82)	344 (80.8)
Family/friend's COVID-19 death			
Yes	144 (9.6)	92 (8.5)	52 (12.1)
No	1362 (90.4)	985 (91.5)	377 (87.9)
**Admission-related variables**			
Secondary School Certificate GPA			
Poor (<4.5)	300 (22.7)	219 (23.7)	81 (20.5)
Moderate (4.5–4.99)	481 (36.4)	351 (38)	130 (32.8)
High (5)	539 (40.8)	354 (38.3)	185 (46.7)
Higher Secondary School Certificate GPA			
Poor (<4.5)	146 (11.1)	91 (9.9)	55 (13.9)
Moderate (4.5–4.99)	383 (29.1)	257 (27.9)	126 (31.9)
High (5)	786 (59.8)	572 (62.2)	214 (54.2)
Coached by professional or coaching centre			
Yes	1036 (73.1)	812 (80.6)	224 (54.6)
No	381 (26.9)	195 (19.4)	186 (45.4)
Desired institute/department for admission			
Varsity	1100 (76.4)	801 (78.3)	299 (71.9)
Medical	217 (15.1)	146 (14.3)	71 (17.1)
Engineering	89 (6.2)	64 (6.3)	25 (6)
Agriculture	33 (2.3)	12 (1.2)	21 (5)
Satisfied with previous mock tests			
Yes	508 (37.5)	365 (37.6)	143 (37.2)
No	848 (62.5)	607 (62.8)	241 (62.8)
Average monthly expenditure (BDT)			
<5000	203 (19.9)	135 (18.9)	68 (22.3)
5000–10 000	601 (58.9)	421 (58.8)	180 (59)
>10 000	217 (21.3)	160 (14.7)	57 (18.7)
Educational background			
Science	886 (58.6)	601 (55.6)	285 (66.3)
Arts	516 (34.1)	405 (37.5)	111 (25.8)
Commerce	109 (7.2)	75 (6.9)	34 (7.9)
**Mental health problems**			
Depression			
No	704 (46.2)	526 (48.3)	178 (41.0)
Yes	819 (53.8)	563 (51.7)	256 (59.0)
Anxiety			
No	1018 (66.8)	759 (69.7)	259 (59.7)
Yes	505 (33.2)	330 (30.3)	175 (40.3)
Burnout			
No	999 (65.6)	727 (66.8)	272 (62.7)
Yes	524 (34.4)	362 (33.2)	162 (37.3)

BDT, Bangladeshi Taka; GPA, grade point average.

### Prevalence of suicidal behaviours

**Fig. 1:**
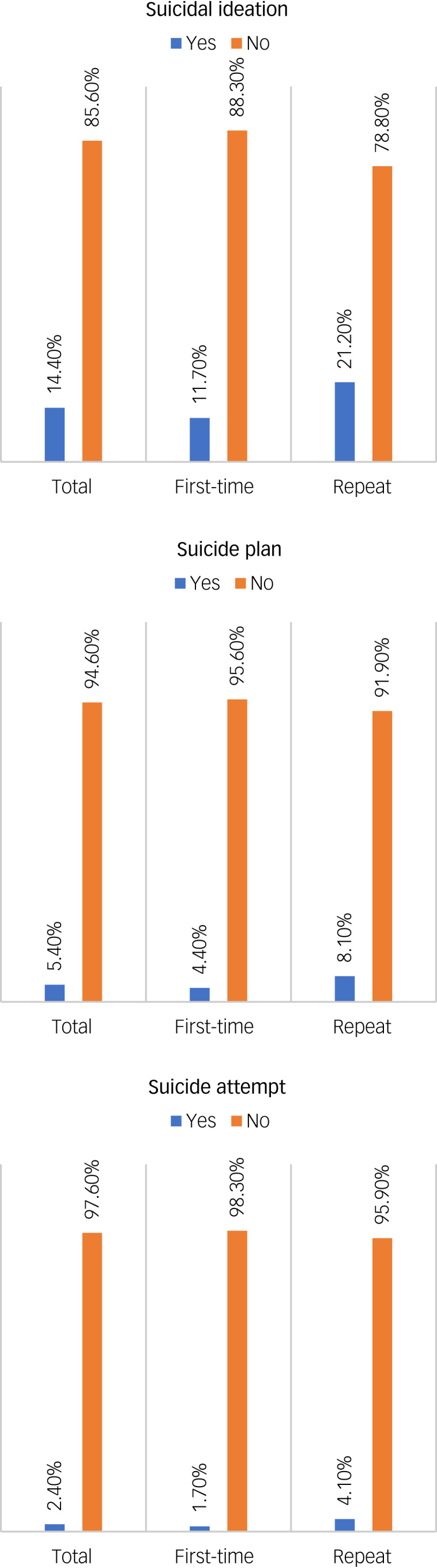
Prevalence of suicidal behaviours based on student status.

Figure 1 shows the prevalence of different suicidal behaviours with respect to student status. In this study, 14.4% of participants reported suicidal ideation, with repeat test-taking students more likely to report suicidal ideation than first-time test takers (21.2% *v.* 11.7%; *χ*^2^ = 22.921, *P* < 0.001). In addition, 5.4 and 2.4% of the students in the total sample had made suicide plans or suicide attempts, respectively. Repeat test-taking students were more likely than first-time test takers to be suicide planners (8.1% *v.* 4.4%; *χ*^2^ = 8.053, *P* = 0.005) and attempters (4.1% *v.* 1.7%; *χ*^2^ = 8.368, *P* = 0.004) (Fig. 1).

### Associations between the studied variables and suicidal ideation

Table 2 presents the relationships between the studied variables and suicidal ideation. Although gender was not statistically associated with suicidal ideation, participants from rural areas reported higher suicidal ideation rates than those from urban areas, and this association was statistically significant both for the total sample (*χ*^2^ = 10.548, *P* = 0.001) and among first-time test-taking students (*χ*^2^ = 7.183, *P* = 0.007). Although the cigarette smokers in the total sample were more likely to have thought about suicide than the non-smokers, drug use status was significantly associated with suicidal thoughts in any of the three groups (Table 2).

**Table 2:** Distribution of suicidal ideation among university entrance test-taking students

Variables	Total, *n* (%)			First-time test taker, *n* (%)			Repeat test taker, *n* (%)		
	Yes (%)	*χ^2^*-test value	*P*-value	Yes (%)	*χ^2^*-test value	*P*-value	Yes (%)	*χ^2^*-test value	*P*-value
**Sociodemographic variables**									
Gender									
Female	43 (12.1)	1.867	0.172	29 (11.0)	136	0.712	14 (15.4)	2.330	0.127
Male	176 (15.1)			98 (11.9)			78 (22.7)		
Permanent residence									
Rural	142 (12.6)	10.548	0.001	84 (10.2)	7.183	0.007	58 (19.1)	1.982	0.159
Urban	73 (19.4)			41 (16.4)			32 (25.2)		
Religion									
Muslim	184 (14.2)	2.192	0.139	111 (11.8)	0.046	0.831	73 (20.2)	2.872	0.090
Other	35 (18.2)			16 (12.5)			19 (29.7)		
Family type									
Nuclear	152 (13.6)	1.986	0.159	87 (10.8)	1.981	0.159	65 (21.2)	0.028	0.866
Joint	58 (16.7)			33 (14.1)			25 (21.9)		
Monthly income (BDT)									
<15 000	49 (13.1)	5.227	0.073	28 (10.5)	2.015	0.365	21 (19.3)	3.146	0.207
15 000–3000	58 (15.1)			35 (13.3)			23 (19.2)		
>30 000	53 (19.6)			26 (14.9)			27 (28.1)		
Cigarette smoking									
Yes	31 (20.4)	4.449	0.035	16 (16.5)	2.171	0.141	15 (27.3)	1.248	0.264
No	186 (14.0)			109 (11.4)			77 (20.6)		
Drug usage status									
Yes	18 (30.5)	12.507	<0.001	9 (22.5)	4.543	0.033	9 (22.5)	7.920	0.005
No	198 (13.9)			116 (11.4)			82 (20.2)		
**COVID-19 related information**									
Personal COVID-19 infection									
Yes	37 (29.4)	24.631	<0.001	22 (25.0)	16.257	<0.001	15 (39.5)	8.148	0.004
No	180 (13.1)			104 (10.6)			76 (19.6)		
Family/friend's COVID-19 infection									
Yes	57 (20.7)	10.103	0.001	33 (17.1)	6.245	0.012	24 (29.3)	3.530	0.060
No	162 (13.2)			94 (10.7)			68 (19.8)		
Family/friend's COVID-19 death									
Yes	39 (27.1)	21.038	<0.001	20 (21.7)	9.815	0.002	19 (36.5)	8.641	0.003
No	177 (13.0)			106 (10.8)			71 (18.8)		
**Admission-related variables**									
Secondary School Certificate GPA									
Poor (<4.5)	41 (13.7)	5.410	0.067	23 (10.5)	5.324	0.070	18 (22.2)	0.170	0.918
Moderate	62 (12.9)			35 (10.0)			27 (20.8)		
High (5)	96 (17.8)			54 (15.3)			42 (22.7)		
Higher Secondary School Certificate GPA									
Poor (<4.5)	23 (15.8)	0.400	0.819	13 (14.3)	1.582	0.453	10 (18.2)	0.486	0.784
Moderate	54 (14.1)			26 (10.1)			28 (22.2)		
High (5)	121 (15.4)			73 (12.8)			48 (22.4)		
Coached by professional or coaching centre									
No	142 (13.7)	2.512	0.113	94(11.6)	0.234	0.629	48 (21.4)	<0.001	0.985
Yes	65 (17.1)			25 (1)2.8			40(21.5)		
Desired institute/department for admission									
Varsity	146 (13.3)	6.676	0.083	84 (10.5)	7.413	0.060	62 (20.7)	4.566	0.206
Medical	40 (18.4)			26 (17.8)			14 (19.7)		
Engineering	12 (13.5)			10 (15.6)			2 (8.0)		
Agriculture	8(24.2)			1(8.3)			7 (33.3)		
Satisfied with previous mock tests									
Yes	63 (12.4)	3.354	0.067	42 (11.5)	0.476	0.490	21 (14.7)	4.457	0.035
No	136 (16.0)			79 (13.0)			57 (23.7)		
Average monthly expenditure (BDT)									
<5000	21 (10.3)	6.316	0.043	10 (7.4)	5.972	0.050	11 (16.2)	2.496	0.287
5000–10 000	99 (16.5)			53 (12.6)			46 (25.6)		
>10 000	41 (18.9)			27 (16.9)			14 (24.6)		
Educational background									
Science	144 (16.3)	5.424	0.066	81 (13.5)	4.404	0.111	63 (22.1)	0.993	0.609
Arts	61 (11.8)			37 (9.1)			24 (21.6)		
Commerce	14 (12.8)			9 (12.0)			5 (14.7)		
**Mental health problems**									
Depression									
No	46 (6.5)	65.448	<0.001	32 (6.1)	30.733	<0.001	14 (7.9)	32.114	<0.001
Yes	173 (21.1)			95 (16.9)			78 (30.5)		
Anxiety									
No	98 (9.6)	56.329	<0.001	63 (8.3)	27.475	<0.001	35 (13.5)	22.707	<0.001
Yes	121 (24.0)			64 (19.4)			57 (32.6)		
Burnout									
No	127 (12.7)	6.552	0.010	83 (11.4)	0.128	0.721	44 (16.2)	11	0.001
Yes	92 (17.6)			44 (12.2)			48 (29.6)		

BDT, Bangladeshi Taka; GPA, grade point average.

About 29.4% of the total sample who had been infected with COVID-19 reported suicidal ideation (*χ*^2^ = 24.631, *P* < 0.001; 13.1%, compared with those who had not been infected), whereas 25% and 39.5% of first-time test takers (*v.* 10.6%; *χ*^2^ = 16.257, *P* < 0.001) and repeat test takers (*v.* 19.6%; *χ*^2^ = 8.148, *P* = 0.004) had suicidal ideation, respectively. Similarly, those students who had experienced someone they had a close relationship being infected with or dying because of the virus were more prone to suicidal ideation (Table 2).

Concerning factors related to the admission test itself, the monthly expenditure of the students while being prepared for the test was significantly associated with suicidal ideation in the total sample. However, students suffering from any mental health problem (i.e. depression, anxiety or burnout) were more likely to have suicidal ideation. That is, depressed participants had higher prevalence of suicidal ideation in the total sample (*χ*^2^ = 65.448, *P* < 0.001; 21.1% *v.* 6.5% compared with those without depression) and in both the first-time and repeat groups (*χ*^2^ = 30.733, *P* < 0.001; and *χ*^2^ = 32.114, *P* < 0.001). Similarly, anxious participants reported higher suicidal ideation rates in the total sample (*χ*^2^ = 56.329, *P* < 0.001 compared with those without anxiety) and among both first-timers and repeaters (*χ*^2^ = 27.475, *P* < 0.001; and *χ*^2^ = 22.707, *P* < 0.001). In addition, students with burnout had more suicidal ideation in the total sample (*χ*^2^ = 6.552, *P=* 0.010 compared with those without burnout) and among repeat test-takers (*χ*^2^ = 11, *P* = 0.001) (Table 2).

### Factors associated with suicidal ideation

Regarding sociodemographic factors, urban participants (odds ratio (OR) = 1.66, 95% CI = 1.22–2.27, *P* = 0.001), cigarette smokers (OR = 1.57, 95% CI = 1.03–2.40, *P* = 0.036) and drug users (OR = 2.71, 95% CI = 1.52–4.81, *P* = 0.001) were at higher risk of suicidal ideation in the total sample. Among the first-time test takers, coming from rural areas and using drugs were risk factors for suicidal ideation, whereas only drug use was a risk factor for suicidal ideation among the repeaters. Among the total participants, there was a significant difference in the risk of suicidality based on student status. Repeat test takers had a higher risk of suicidal ideation compared with first-timers, with an OR of 2.03 (95% CI = 1.51–2.73, *P* < 0.001) (Table 3).

**Table 3:** Regression analysis of suicidal ideation among students taking university entrance tests

Variables	Total sample		First-time test taker		Repeat test taker	
	OR (95% CI)	*P*-value	OR (95% CI)	*P*-value	OR (95% CI)	*P*-value
**Sociodemographic variables**						
Gender						
Male	1.28 (0.89–1.83)	0.173	1.08 (0.70–1.68)	0.712	1.61 (0.86–3.01)	0.130
Female	Reference		Reference		Reference	
Permanent residence						
Urban	1.66 (1.22–2.27)	0.001	1.72 (1.15–2.58)	0.008	1.42 (0.87–2.32)	0.160
Rural	Reference		Reference		Reference	
Religion						
Muslim	0.74 (0.49–1.10)	0.140	0.94 (0.53–1.64)	0.831	0.60 (0.33–1.08)	0.093
Other	Reference		Reference		Reference	
Family type						
Nuclear	0.78 (0.56–1.09)	0.159	0.73 (0.47–1.13)	0.161	0.95 (0.56–1.61)	0.866
Joint	Reference		Reference		Reference	
Monthly income (BDT)						
<15 000	0.61 (0.40–0.94)	0.075	0.67 (0.37–1.18)	0.368	0.61 (0.31–1.17)	0.211
15 000–3000	0.73 (0.48–1.10)		0.87 (0.50–1.51)		0.60 (0.32–1.14)	
>30 000	Reference		Reference		Reference	
Cigarette smoking						
Yes	1.57 (1.03–2.40)	0.036	1.53 (0.86–2.71)	0.143	1.44 (0.75–2.74)	0.266
No	Reference		Reference		Reference	
Drug usage						
Yes	2.71 (1.52–4.81)	0.001	2.25 (1.04–4.85)	0.038	3.54 (1.39–9.00)	0.008
No	Reference		Reference		Reference	
**COVID-19 related information**						
Personal COVID-19 infection						
Yes	2.75 (1.82–4.16)	<0.001	2.82 (1.67–4.76)	<0.001	2.67 (1.33–5.37)	0.006
No	Reference		Reference		Reference	
Family/friend's COVID-19 infection						
Yes	1.71 (1.22–2.39)	0.002	1.11 (0.48–2.53)	0.013	1.01 (0.31–3.28)	0.062
No	Reference		Reference		Reference	
Family/friend's COVID-19 death						
Yes	2.48 (1.66–3.71)	<0.001	2.30 (1.34–3.93)	0.002	2.48 (1.33–4.61)	0.004
No	Reference		Reference		Reference	
**Admission-related variables**						
Secondary School Certificate GPA						
Poor (<4.5)	0.73 (0.49–1.08)	0.068	0.65 (0.38–1.09)	0.072	0.97 (0.52–1.82)	0.918
Moderate	0.68 (0.48–0.96)		0.61 (0.39–0.96)		0.89 (0.51–1.54)	
High (5)	Reference		Reference		Reference	
Higher Secondary School Certificate GPA						
Poor (<4.5)	1.02 (0.63–1.67)	0.819	1.13 (0.60–2.15)	0.455	0.76 (0.36–1.63)	0.785
Moderate	1.90 (0.63–1.27)		0.76 (0.47–1.23)		0.98 (0.58–1.67)	
High (5)	Reference		Reference		Reference	
Coached by professional or coaching centre						
No	1.29 (0.94–1.78)	0.114	1.12 (0.70–1.80)	0.629	1.00 (0.62–1.61)	0.985
Yes	Reference		Reference		Reference	
Desired institute/department for admission						
Varsity	0.47 (0.21–1.08)	0.088	1.28 (0.16–10.10)	0.064	0.52 (0.20–1.35)	0.237
Medical	0.70 (0.29–1.68)		2.38 (0.29–19.28)		0.49 (0.16–1.44)	
Engineering	0.48 (0.17–1.32)		2.03 (0.23–17.58)		0.17 (0.03–0.95)	
Agriculture	Reference		Reference			
Satisfied with previous mock tests						
No	1.34 (0.97–1.80)	0.068	1.15 (0.77–1.71)	0.491	1.80 (1.03–3.12)	0.036
Yes	Reference		Reference		Reference	
Average monthly expenditure (BDT)						
<5000	0.49 (0.28–0.87)	0.046	0.39 (0.18–0.84)	0.056	0.59 (0.24–1.43)	0.294
5000–10 000	0.41 (0.56–1.26)		0.70 (0.42–1.17)		1.05 (0.52–2.10)	
>10 000	Reference		Reference		Reference	
Educational background						
Science	1.31 (0.73–2.37)	0.068	1.14 (0.54–2.38)	0.113	1.64 (0.61–4.42)	0.614
Arts	0.91 (0.48–1.69)		0.73 (0.34–1.59)		1.60 (0.55–4.57)	
Commerce	Reference		Reference		Reference	
**Mental health problems**						
Depression						
Yes	3.83 (2.71–5.39)	<0.001	3.13 (2.05–4.77)	<0.001	5.13 (2.79–9.42)	<0.001
No	Reference		Reference		Reference	
Anxiety						
Yes	2.95 (2.21–3.96)	<0.001	2.65 (1.82–3.86)	<0.001	3.09 (1.92–4.97)	<0.001
No	Reference		Reference		Reference	
Burnout						
Yes	1.46 (1.09–1.95)	0.011	1.07 (0.72–1.58)	0.721	2.18 (1.36–3.48)	0.032
No	Reference		Reference		Reference	

BDT, Bangladeshi Taka; GPA, grade point average.

All of the COVID-19-related variables were significant risk factors for suicidal ideation. Among all participants, a 2.75-fold higher risk of experiencing suicidality was reported among those who had been infected with COVID-19 compared with those who had not; similarly, there were 2.82-fold and 2.67-fold increases in risk for the first-timers and the repeaters, respectively. In addition, both COVID-19 infection and death among family and/or friends were risk factors in all three groups, except for COVID-19-related death of family members and/or friends in the repeat test-taking group. Among the total participants, depressed and anxious participants had a threefold higher risk of suicidal ideation than those without depression or anxiety (OR = 3.83, 95% CI = 2.71–5.39, *P* < 0.001 for depression; OR = 2.95, 95% CI = 2.21–3.96, *P* < 0.001 for anxiety). Participants with burnout who were taking an exam for the second time had a significantly higher risk of suicidal ideation than those without burnout (OR = 2.18, 95% CI = 1.36–3.48, *P* = 0.032) (Table 3).

Adjusted models of the factors associated with suicidal ideation in the total sample and both test-taking student groups are provided in the Supplementary file available at https://doi.org/10.1192/bjo.2023.526.

### Suicidal ideation across districts

**Fig. 2:**
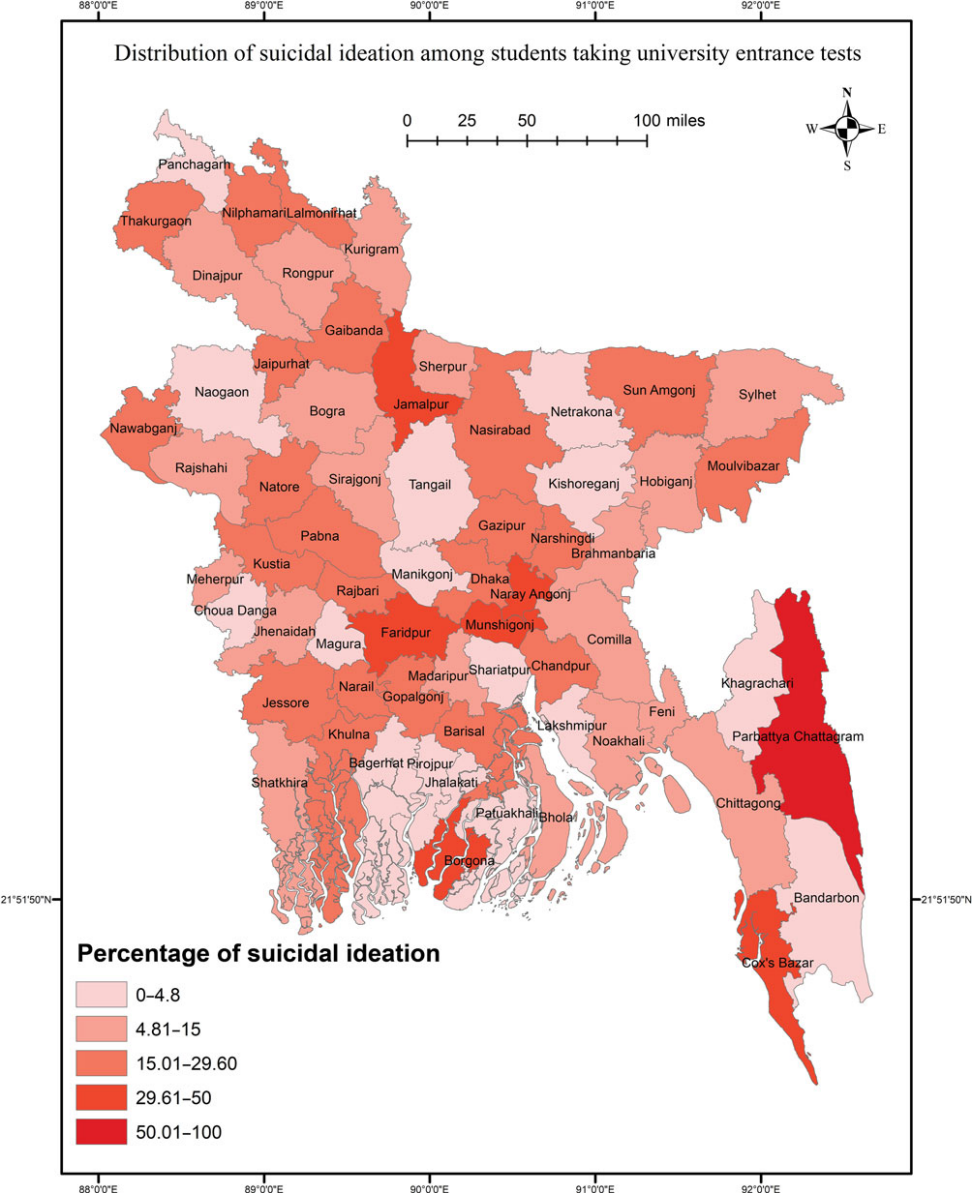
Geographic Information System-based distribution of suicidal ideation among students taking university entrance test.

**Fig. 3:**
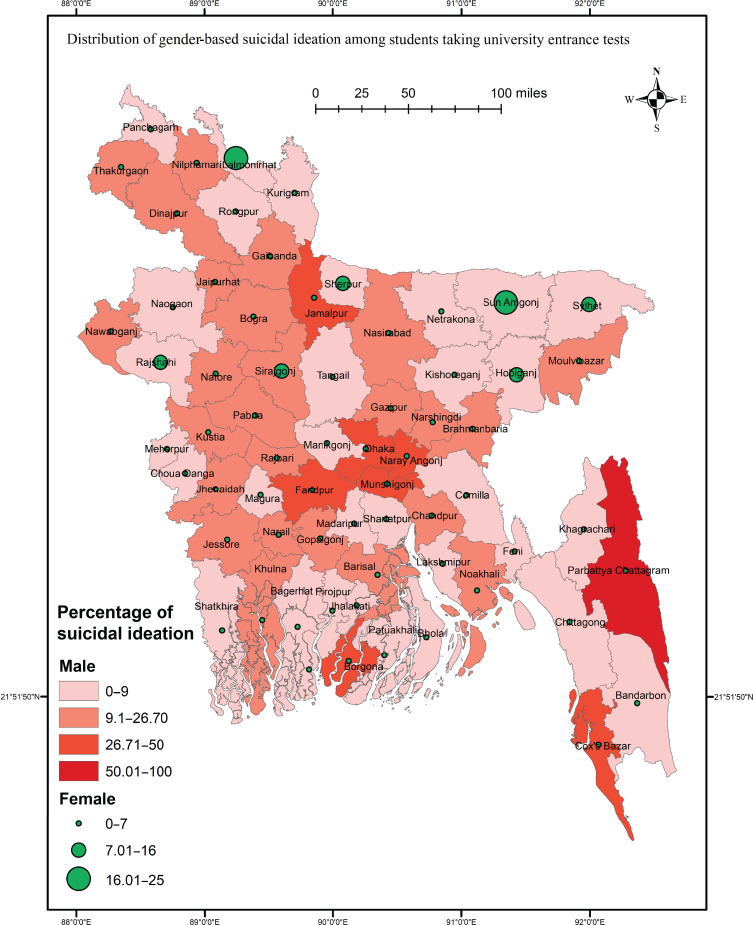
Geographic Information System-based distribution of suicidal ideation by gender.

**Fig. 4:**
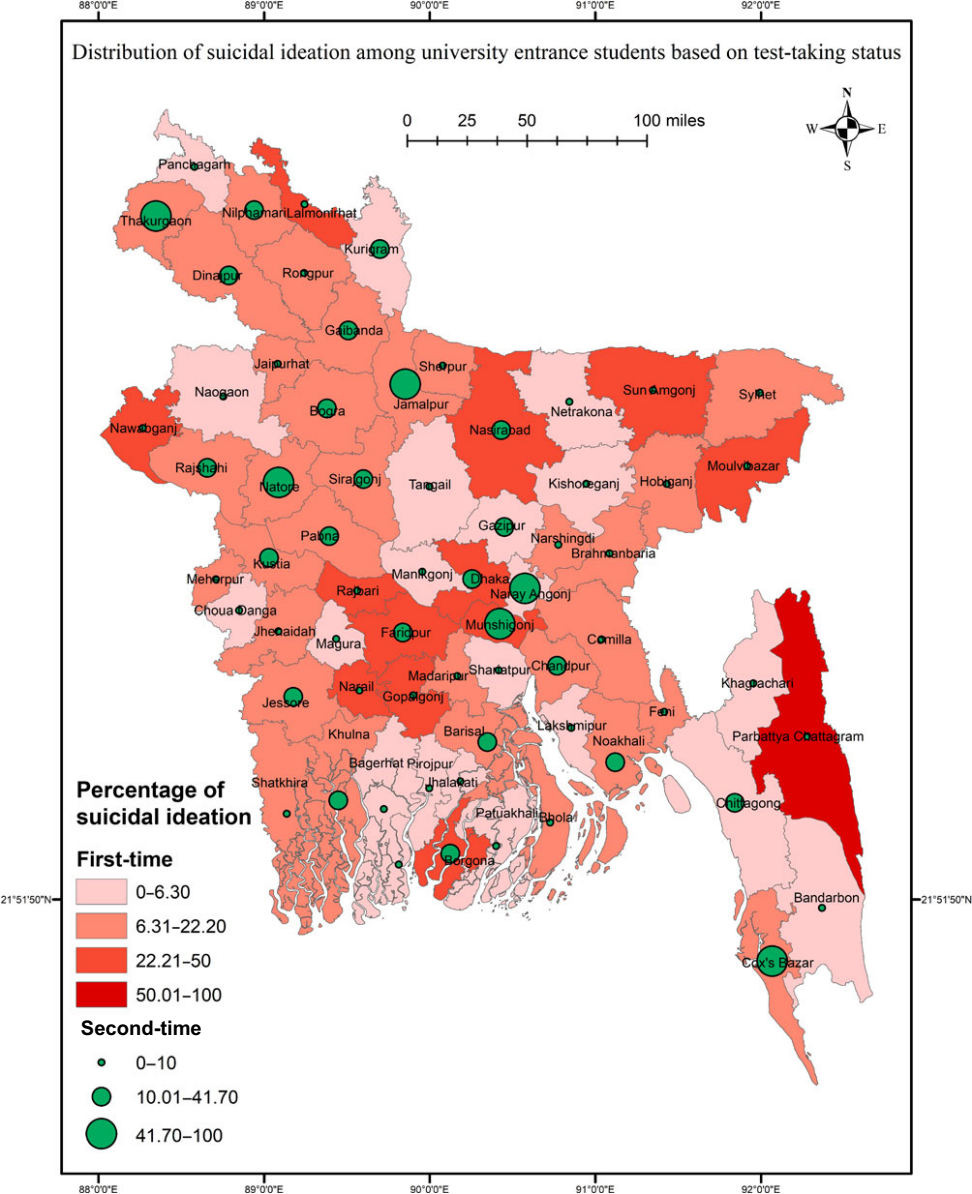
Geographic Information System-based distribution of suicidal ideation by student status.

The nationwide distribution of suicidal ideation was determined using spatial analysis. The results suggested a significant association between district and suicidal ideation (χ^2^ = 97.409, *P* = 0.002). The prevalence of suicidal ideation was high in some northern districts such as Thakurgaon, Nilphamari and Jamalpur. The Chittagong Hill Tract area showed high suicidal ideation, whereas lower suicidal ideation was reported in Tangail, Naogaon, Kishoreganj and Netrakona (Fig. 2). Of the gender-based distribution, suicidal ideation only differed significantly among regions for males (χ^2^ = 95.940, *P* = 0.001), whereas high prevalence rates was reported for Faridpur, Munshigonj, Naryangonj and Jamalpur (Fig. 3). The rate of suicidal ideation did not vary significantly by student status (*P* > 0.05), though the rate was high in Dhaka, Narayangonj, Faridpur and Mymensingh (Fig. 4).

## Discussion

This study attempts to determine the magnitude of suicidal behaviours among students taking university entrance tests after the COVID-19 pandemic in Bangladesh. District-wise rates of the suicidal ideation were visualised using GIS mapping. According to this study, 14.4% of students taking entrance tests reported experiencing suicidal ideation in the past year, whereas 5.4% had made suicide plans and 2.4% had made suicide attempts. Notably, repeat test takers exhibited a higher prevalence of suicidal behaviours than first-time test takers (21.2% *v.* 11.7% for suicidal ideation, 8.1% *v.* 4.4% for suicide plans and 4.1% *v.* 1.7% for suicide attempts). Several factors were found to be associated with suicidal ideation, including residing in an urban area, smoking, drug use, COVID-19 infection or having a close relationship with someone infected, experiencing the death of a family member or friend owing to the virus, and suffering from mental disorders such as depression, anxiety and burnout. The GIS-based distribution revealed significant variations in the prevalence of suicidal ideation across different districts, with higher rates observed in northern regions and Hill Tract areas. Whereas a significant difference in suicidal ideation distribution by region was observed among males in the GIS mapping, this was not the case for females. Although districts near the capital displayed higher rates of suicidal ideation, no significant differences were found based on student status.

Given the stressors and disruptions caused by the COVID-19 pandemic,^10,11^ it was anticipated that the prevalence of suicidal behaviours would increase compared with those reported by a previous study conducted among a similar student population in 2019.^3^ Surprisingly, the current study reported a lower rate of suicidal ideation, with 14.4% of test-taking students experiencing such thoughts, as opposed to the previous study's rate of 17.7%.^3^ The exact reasons for this unexpected result remain uncertain and warrant further investigation. However, it is noteworthy that the prevalence rates for suicide plans and attempts in this study were 5.4% and 2.4%, respectively; these rates were lower than those reported by the previous study (8.0% and 2.5%, respectively).^3^ Still, the substantial number of suicidal behaviours existing in this population is a deeply concerning trend, highlighting the critical need for targeted interventions and support to address the escalating risk of self-harm among students taking university entrance tests during these challenging times.

In the present study, significant differences were observed in the distribution of suicidal behaviours based on student status. Repeat test-taking students reported higher rates of suicidal behaviours compared with first-time test-takers. Specifically, the prevalence of suicidal ideation was 21.2% among repeat test takers, compared with 11.7% among first-time test takers in this study. Similarly, the rates for suicide plans were 4.4 and 6.6%, whereas those for suicide attempts were 9.9 and 6.1%, respectively. It is worth noting that most universities in Bangladesh allow students to take the entrance test up to two times. Those who have previously failed the test and are attempting it for the final time may experience greater psychological distress than those taking it for the first time. There is an ongoing debate regarding the potential success of repeat test takers, as these students have more time to prepare and may feel more confident and psychologically stable for the exam. Evidently, repeat test-taking students in the previous study reported higher prevalence rates of psychological problems, including depression (53.7% *v.* 41.9%), anxiety (33.9% *v.* 23.7%) and burnout (48% *v.* 39.2%), compared with those students taking the test for the first time.^3^ Similarly, the previous study reported that suicidal ideation was significantly more prevalent among repeat test takers compared with first-time test takers (20.7% *v.* 14.6%).^3^ These findings align with a suicide case-series study conducted during the COVID-19 pandemic, which reported that academic failure accounted for one in every ten suicides among Bangladeshi students.^19^ In the context of students taking entrance tests, repeat test takers appear to be at higher risk of suicidality and actual suicide.

Gender-based differences appear to have a significant role in suicidality. The previous study among test-taking students revealed that female students had a twofold higher risk of suicidal ideation.^8^ However, in the present study, no significant difference was found between gender and suicidal ideation. This unexpected result could be attributed to several unknown factors. It is worth noting that the sampling proportions for males and females were not identical between the two studies, which might have influenced the rates of suicidality. Further research should be conducted using more rigorous sampling methods to elucidate this relationship, with a particular focus on maintaining gender-based equality during participant recruitment.

On the other hand, this study identified urban residency as a risk factor for suicidal ideation, consistent with the previous study's findings.^3^ Furthermore, students with unhealthy lifestyles, including drug use and cigarette smoking, were found to have a higher risk of suicidal behaviour in this study. A previous study among adolescents found that early initiation of hard drug use among males and engaging in risky behaviours such as smoking, alcohol consumption and drug use among females were strongly associated with suicidality.^20^ Another study reported that risky sexual behaviours, drug involvement and tobacco use were predictive factors for suicide attempts.^21^ Therefore, when implementing suicide prevention programmes, it is crucial to consider factors related to unhealthy lifestyles.

COVID-19-related experiences such as personal infection, infection among family members or friends, and deaths of individuals with close relationships to the participant owing to the infection were significant factors associated with suicidal ideation. Throughout the COVID-19 pandemic, individuals have been confronted with the unwanted fear of contracting the virus, and this has been linked to suicide.^22,23^ Furthermore, a study has reported cases of suicide directly related to COVID-19 infection.^22^ A recent systematic review of studies conducted in Bangladesh during the pandemic highlighted factors associated with suicidal events, including a lack of knowledge about COVID-19, residing in highly infected regions, fear of infection, personal infection with the virus and experiencing the death of someone close owing to the infection.^10^ Consequently, students who have personally experienced COVID-19 infection or have faced mortality in their close relationships are at a heightened risk of suicidal ideation; thus, these students should be a focus when mental health support programmes are implemented.

According to a recent study, approximately 9% of suicides among Bangladeshi students during the COVID-19 pandemic could be attributed to mental health problems.^19^ Various factors, including emotional issues, family conflicts, relationship complexities and sexual problems which are closely associated with mental disorders,^24,25^ account for the remaining suicides.^19^ Therefore, it was not surprising to observe higher rates of suicidality among students with mental health problems, as was also found in the previous study. The previous study reported a twofold higher risk of suicidality among students experiencing burnout, indicating high-stress levels and reduced academic efficacy.^3^ Furthermore, individuals who were depressed or anxious in the previous study had a fourfold higher risk of suicidal ideation,^3^ consistent with the present study's findings. Since the onset of the COVID-19 pandemic, individuals have been reported to experience mental and emotional problems. A systematic review of studies conducted during the pandemic revealed that approximately half of the Bangladeshi population suffered from mental health problems. The pooled prevalence rates were 47% for depression, and 47% for anxiety, and 44% for stress.^11^ Such circumstances have undoubtedly affected the mental health of students presenting for university entrance tests, as they face uncertainty regarding their education and future prospects due to the pandemic-related restrictions and disruptions.

The GIS-based distribution analysis in this study revealed significant variations in the prevalence of suicidal ideation across different districts of the country. Test-taking students from northern regions such as Thakurgaon, Nilphamari, Jamalpur and Chittagong Hill Tract exhibited higher rates of suicidal ideation. These regions of Bangladesh are known to have higher poverty rates and limited educational opportunities compared with other areas.^26^ The Chittagong Hill Tract region also faces challenges regarding access to modern facilities, including education and transportation.^27^ These factors may contribute to the higher prevalence of suicidal ideation in these regions. When examining the gender-based distribution of suicidal ideation by district, a significant association only for males was found. Specifically, males from districts including Faridpur, Munshigonj, Naryangonj and Jamalpur exhibited higher rates of suicidal ideation than males in other regions. On the other hand, the GIS mapping did not show any significant association between student status and suicidal ideation. However, districts near the capital, including Dhaka, Narayangonj, Faridpur and Mymensingh, had higher rates of suicidal ideation than other regions.

This study presents valuable evidence regarding suicidal behaviour and associated factors among students taking university entrance tests in the context of the COVID-19 pandemic. However, certain limitations should be acknowledged. First, the study design was cross-sectional, which limited the ability to establish causal relationships between variables. Conducting longitudinal studies would provide more robust evidence in this regard. Second, the sampling distribution could be considered a limitation as the majority of participants belonged to the first-time test taker group, although a *post hoc* analysis was performed to address potential biases. Another limitation was the reliance on self-reporting survey methods to collect data. Although the research team provided briefings about the questionnaire to ensure accurate information, self-reporting introduces the possibility of social desirability bias; memory recall bias is also a common concern in self-report surveys. It is important to consider these limitations when interpreting the results of the study, and future research should aim to address these issues using more diverse and representative sampling methods, longitudinal designs and complementary data collection techniques to minimise biases.

In conclusion, the findings of this study underscore the urgent need for comprehensive measures to address the prevalence of suicidal behaviours among students taking university entrance tests in Bangladesh, especially in the context of the COVID-19 pandemic. The recommendations derived from the study's findings include enhancing tailored mental health services and support for repeat test takers, and considering other risk factors such as unhealthy lifestyles, COVID-19 exposure and mental health problems among test-taking students. Developing and implementing educational programmes to raise awareness, combat stigma and encourage help-seeking behaviours are crucial. Using GIS-based mapping to analyse the distribution of suicidal ideation can provide valuable insights for targeted suicide-prevention efforts. Moreover, it is imperative to allocate resources towards improving educational opportunities and infrastructure in economically disadvantaged regions, particularly in northern areas and Chittagong Hill Tract, to address disparities and safeguard mental well-being. By implementing these recommendations, stakeholders can pro-actively address the mental health challenges faced by students taking university entrance tests and create a supportive environment that promotes their overall well-being.

## Data Availability

The data that support the findings of this study are available on request from the corresponding author (R.N.).

## Supplementary material

For supplementary material accompanying this paper visit http://doi.org/10.1192/bjo.2023.526. click here to view supplementary material

## References

[1] Stallman HM. Psychological distress in university students: a comparison with general population data. Aust Psychol 2010; 45: 249–57.

[2] Faye-Dumanget C, Carré J, le Borgne M, Boudoukha PAH. French validation of the Maslach Burnout Inventory-Student Survey (MBI-SS). J Eval Clin Pract 2017; 23: 1247–51. 28653800 10.1111/jep.12771

[3] Mamun MA, Misti JM, Hosen I, al Mamun F. Suicidal behaviors and university entrance test-related factors: a Bangladeshi exploratory study. Perspect Psychiatr Care 2022; 58: 278–87. 33834493 10.1111/ppc.12783

[4] Sakib N, Islam M, Al Habib MS, Bhuiyan AKMI, Alam MM, Tasneem N, et al. Depression and suicidality among Bangladeshi students: subject selection reasons and learning environment as potential risk factors. Perspect Psychiatr Care 2021; 57: 1150–62. 33135191 10.1111/ppc.12670

[5] Tribune Desk. HSC: girls ahead of boys in securing GPA 5. *Dhaka Tribune*, 30 Jan 2021 (https://archive.dhakatribune.com/feature/education-feature/2021/01/30/hsc-girls-ahead-of-boys-in-securing-gpa-5 [cited 17 May 2023]).

[6] Alamgir M, Rahaman A. HSC pass rate falls. *Daily Star*, 9 Feb 2023 (https://www.thedailystar.net/youth/education/news/hsc-pass-rate-falls-3242756 [cited 17 May 2023]).

[7] Akhter S. 30pc seats in universities, colleges in Bangladesh to remain vacant. *New Age Bangladesh*, 10 Feb 2023 (https://www.newagebd.net/article/194025/30pc-seats-in-universities-colleges-to-remain-vacant [cited 17 May 2023]).

[8] Mamun MA, Safiq MB, Hosen I, al Mamun F. Burnout, does the university entrance test failing attribute? A Bangladeshi exploratory study. PLoS One 2021; 16: e0258100. 10.1371/journal.pone.0258100 PMC8491878 34610010

[9] Fleischmann A, Bertolote JM, Belfer M, Beautrais A. Completed suicide and psychiatric diagnoses in young people: a critical examination of the evidence. Am J Orthopsychiatry 2005; 75: 676–83. 16262523 10.1037/0002-9432.75.4.676

[10] Mamun MA. Suicide and suicidal behaviors in the context of COVID-19 pandemic in Bangladesh: a systematic review. Psychol Res Behav Manag 2021; 14: 695–704. 34113185 10.2147/PRBM.S315760 PMC8185458

[11] Hosen I, Al-Mamun F, Mamun MA. Prevalence and risk factors of the symptoms of depression, anxiety, and stress during the COVID-19 pandemic in Bangladesh: a systematic review and meta-analysis. Glob Ment Health 2021; 8: e47. 10.1017/gmh.2021.49 PMC8794743 35145709

[12] Kroenke K, Spitzer RL, Williams JBW. The PHQ-9: validity of a brief depression severity measure. J Gen Intern Med 2001; 16: 606. 11556941 10.1046/j.1525-1497.2001.016009606.x PMC1495268

[13] Bhuiyan MAH, Griffiths MD, Mamun MA. Depression literacy among Bangladeshi pre-university students: differences based on gender, educational attainment, depression, and anxiety. Asian J Psychiatr 2020; 50: e101944. 10.1016/j.ajp.2020.101944 32106072

[14] Spitzer RL, Kroenke K, Williams JBW, Löwe B. A brief measure for assessing generalized anxiety disorder: the GAD-7. Arch Intern Med 2006; 166: 1092–7. 16717171 10.1001/archinte.166.10.1092

[15] Schaufeli WB, Martinez IM, Pinto AM, Salanova M, Bakker AB. Burnout and engagement in university students: a cross-national study. J Cross Cult Psychol 2002; 33: 464–81.

[16] Campos JADB, Maroco J. [Maslach Burnout Inventory – Student Survey: Portugal-Brazil cross-cultural adaptation]. Rev Saude Publica 2012; 46: 816–24. 23128258 10.1590/s0034-89102012000500008

[17] Dos Santos Boni RA, Paiva CE, De Oliveira MA, Lucchetti G, Fregnani JHTG, Paiva BSR. Burnout among medical students during the first years of undergraduate school: prevalence and associated factors. PLoS One 2018; 13: e0191746. 29513668 10.1371/journal.pone.0191746 PMC5841647

[18] Turecki G, Brent DA. Suicide and suicidal behaviour. Lancet 2016; 387: 1227–39. 26385066 10.1016/S0140-6736(15)00234-2 PMC5319859

[19] Mamun MA, al Mamun M, Hosen I, Ahmed T, Rayhan I, al-Mamun F. Trend and gender-based association of the Bangladeshi student suicide during the COVID-19 pandemic: a GIS-based nationwide distribution. 2021; 69(1): 38–46. 10.1177/00207640211065670 PMC9936165 34961356

[20] Cho H, Hallfors DD, Iritani BJ. Early initiation of substance use and subsequent risk factors related to suicide among urban high school students. Addict Behav 2007; 32: 1628–39. 17210230 10.1016/j.addbeh.2006.11.017 PMC3744891

[21] Shaughnessy L, Doshi SR, Jones SE. Attempted suicide and associated health risk behaviors among native American high school students. J Sch Health 2004; 74: 177–82. 15283499 10.1111/j.1746-1561.2004.tb08217.x

[22] Dsouza DD, Quadros S, Hyderabadwala ZJ, Mamun MA. Aggregated COVID-19 suicide incidences in India: fear of COVID-19 infection is the prominent causative factor. Psychiatry Res 2020; 290: 113145. 10.1016/j.psychres.2020.113145 PMC7832713 32544650

[23] Mamun MA, Griffiths MD. First COVID-19 suicide case in Bangladesh due to fear of COVID-19 and xenophobia: possible suicide prevention strategies. Asian J Psychiatr 2020; 51: 102073. 32278889 10.1016/j.ajp.2020.102073 PMC7139250

[24] Barnhart S, Garcia AR, Karcher NR. Adolescent mental health and family economic hardships: the roles of adverse childhood experiences and family conflict. J Youth Adolesc 2022; 51: 2294–311. 35997913 10.1007/s10964-022-01671-9

[25] Jordan CE, Campbell R, Follingstad D. Violence and women's mental health: the impact of physical, sexual, and psychological aggression. 2010; 6: 607–28. 10.1146/annurev-clinpsy-090209-151437 20192793

[26] World Bank. *Poverty Maps of Bangladesh 2010: Key Findings*. 2010 (http://203.112.218.65:8008/WebTestApplication/userfiles/Image/LatestReports/Poverty_Map_brochure10.pdf [cited 17 May 2023]).

[27] Star Digital Report. Lack of access and remoteness of many communities remains a challenge in CHT. *Daily Star*, 17 Nov 2022 (https://www.thedailystar.net/news/bangladesh/news/lack-access-and-remoteness-many-communities-remains-challenge-cht-3172281 [cited 17 May 2023]).

