# Supplementary material for: Prevalence and factors associated with suicidal ideation among students taking university entrance tests: revisited and a study based on Geographic Information System data
Source: BJPsych Open. 2023 Jul 18;9(4):e129. doi: 10.1192/bjo.2023.526 (PMC10375905; doi:10.1192/bjo.2023.526)
Supplement: Supplementary file 1 [file S2056472423005264sup001.docx]

**Regression analysis of suicidal ideation among university entrance test-taking students (adjusted model)**

| **Variables** | **Total sample** | | **First-time test-taker** | | **Repeat test-taker** | |
| --- | --- | --- | --- | --- | --- | --- |
|  | **AOR; 95% CI** | ***p*-value** | **AOR; 95% CI** | ***p*-value** | **AOR; 95% CI** | ***p*-value** |
| **Sociodemographic variables** | | | | | | |
| **Gender** | | | | | | |
| Male | 0.88 (0.44 – 1.73) | 0.715 | 0.51 (0.22 – 1.14) | 0.102 | 2.70 (0.63 – 11.50) | 0.178 |
| Female | Reference |  | Reference |  | Reference |  |
| **Permanent residence** | | | | | | |
| Urban | 1.41 (0.85– 2.33) | 0.181 | 1.08 (0.55 – 2.12) | 0.816 | 1.70 (0.64 – 4.43) | 0.278 |
| Rural | Reference |  | Reference |  | Reference |  |
| **Religion** | | | | | | |
| Muslim | 0.57 (0.31 – 1.04) | 0.069 | 0.75 (0.34 – 1.64) | 0.474 | 0.17 (0.04 – 0.58) | **0.005** |
| Others | Reference |  | Reference |  | Reference |  |
| **Family type** | | | | | | |
| Nuclear | 0.90 (0.54 – 1.50) | 0.710 | 0.79 (0.41 – 1.52) | 0.497 | 1.15 (0.41 – 3.23) | 0.788 |
| Joint | Reference |  | Reference |  | Reference |  |
| **Monthly income (BDT)** | | | | | | |
| <15000 | 1.12 (0.58 – 2.12) | 0.605 | 1.19 (0.51 – 2.77) | 0.862 | 1.01 (0.28 – 3.56) | 0.062 |
| 15000-3000 | 0.85 (0.46 – 1.55) |  | 1.23 (0.57 – 2.66) |  | 0.31 (0.09 – 1.07) |  |
| >30000 | Reference |  | Reference |  | Reference |  |
| **Cigarette smoking status** | | | | | | |
| Yes | 0.48 (0.18 – 1.25) | 0.134 | 1.53 (0.15 – 1.91) | 0.338 | 0.49 (0.07 – 3.26) | 0.463 |
| No | Reference |  | Reference |  | Reference |  |
| **Drug usage status** | | | | | | |
| Yes | 1.58 (0.46 – 5.38) | 0.463 | 1.69 (0.34 – 8.35) | 0.517 | 1.71 (0.14 – 19.76) | 0.667 |
| No | Reference |  | Reference |  | Reference |  |
| **COVID-19 related information** | | | | | | |
| **Personal COVID-19 infection** | | | | | | |
| Yes | 2.17 (0.99 –4.72) | 0.051 | 3.16 (1.19 – 8.36) | **0.020** | 0.47 (0.07 – 3.08) | 0.438 |
| No | Reference |  | Reference |  | Reference |  |
| **Family/friend’s COVID-19 infection** | | | | | | |
| Yes | 1.01 (0.54 – 1.90) | 0.960 | 1.14 (0.50 – 2.59) | 0.751 | 0.99 (0.30 – 3.23) | 0.986 |
| No | Reference |  | Reference |  | Reference |  |
| **Family/friend’s COVID-19 death** | | | | | | |
| Yes | 1.73 (0.84 – 3.57) | 0.135 | 1.04 (0.38 – 2.80) | 0.933 | 2.55 (0.57 – 11.39) | 0.219 |
| No | Reference |  | Reference |  | Reference |  |
| **Admission-related variables** | | | | | | |
| **Student Status** | | | | | | |
| Second Timer | 1.49 (0.92 – 2.41) | 0.097 | - | - | - | - |
| First timer | Reference |  | - |  | - |  |
| **Secondary School Certificate (SSC) grade point average** | | | | | | |
| Poor (<4.5) | 0.49 (0.22 – 1.06) | 0.142 | 0.41 (0.14 – 1.13) | 0.231 | 0.56 (0.13 – 2.30) | 0.469 |
| Moderate | 0.63 (0.35 – 1.12) |  | 0.74 (0.36 – 1.52) |  | 0.49 (0.15 – 1.56) |  |
| High (5) | Reference |  | Reference |  | Reference |  |
| **Higher Secondary Certificate (HSC) grade point average** | | | | | | |
| Poor (<4.5) | 1.09 (0.43 – 2.77) | 0.613 | 1.25 (0.36 – 4.34) | 0.543 | 1.63 (0.29 – 8.96) | 0.851 |
| Moderate | 1.35 (0.73 –252) |  | 1.56 (0.70 – 3.48) |  | 1.10 (0.33 – 3.73) |  |
| High (5) | Reference |  | Reference |  | Reference |  |
| **Coached by professional coaching centers** | | | | | | |
| No | 1.10 (0.64 – 1.89) | 0.721 | 1.11 (0.51 – 2.40) | 0.788 | 0.71 (0.28 – 1.79) | 0.470 |
| Yes | Reference |  | Reference |  | Reference |  |
| **Desired institute/department for admission** | | | | | | |
| Varsity | 0.64 (0.17 – 2.40) | 0.674 | 1.07 (0.09 – 11.87) | 0.246 | 0.35 (0.04 – 2.71) | 0.171 |
| Medical | 0.92 (0.24 – 3.53) |  | 2.42 (0.21 – 26.95) |  | 0.25 (0.02 – 2.24) |  |
| Engineering | 0.66 (0.15– 2.78) |  | 2.11 (0.18 – 24.08) |  | 0.02 (0.001 – 0.67) |  |
| Agriculture | Reference |  | Reference |  |  |  |
| **Satisfied with previous mock tests** | | | | | | |
| No | 1.85 (1.10 – 3.10) | **0.019** | 1.29 (0.67 – 2.49) | 0.434 | 5.53 (1.94 – 15.75) | **0.001** |
| Yes | Reference |  | Reference |  | Reference |  |
| **Average monthly expenditure (BDT)** | | | | | | |
| <5,000 | 0.93 (0.43 – 2.03) | 0.848 | 0.81 (0.30 – 2.16) | 0.921 | 0.84 (0.18 – 3.81) | 0.703 |
| 5000-10,000 | 1.10 (0.62 – 1.97) |  | 0.93 (0.46 – 1.89) |  | 1.34 (0.39 – 4.53) |  |
| >10,000 | Reference |  | Reference |  | Reference |  |
| **Educational background** | | | | | | |
| Science | 0.89 (0.35 – 2.23) | 0.632 | 0.73 (0.22 – 2.38) | 0.512 | 1.15 (0.19 – 6.93) | 0.982 |
| Arts | 1.19 (0.46 – 3.03) |  | 1.15 (0.35 – 3.76) |  | 1.06 (0.16 – 6.95) |  |
| Commerce | Reference |  | Reference |  | Reference |  |
| **Mental health problems** | | | | | | |
| **Depression** | | | | | | |
| Yes | 2.69 (1.46 – 4.97) | **0.001** | 2.13 (1.03 – 4.40) | **0.041** | 8.51 (2.00 – 36.17) | **0.004** |
| No | Reference |  | Reference |  | Reference |  |
| **Anxiety** | | | | | | |
| Yes | 1.65 (0.99 – 2.73) | 0.050 | 1.78 (0.93 – 3.40) | 0.081 | 1.72 (0.64 – 4.57) | 0.277 |
| No | Reference |  | Reference |  | Reference |  |
| **Burnout** | | | | | | |
| Yes | 0.93 (0.58 – 1.49) | 0.774 | 0.74 (0.39 – 1.40) | 0.357 | 1.59 (0.66 – 3.84) | 0.299 |
| No | Reference |  | Reference |  | Reference |  |

The results suggested that being not satisfied with previous mock tests increased the risk of suicidal ideation (OR= 1.85, 95% CI=1.10 – 3.10, *p*=0.019). In addition, depressed participants were at 2.69 times higher risk of suicidal ideation than those who were not depressed (OR=2.69, 95% CI=1.46 – 4.97, *p*=0.001). For the first-time test takers, personal COVID-19 infection (OR=3.16, 95% CI=1.19 – 8.36, *p*=0.020) and suffering from depression (OR=2.13, 95% CI=1.03 – 4.40, *p*=0.041) increased the risk of suicidal ideation. For repeat test-takers, being not satisfied with previous mock tests (OR=3.16, 95% CI=1.19 – 8.36, *p*=0.020) and suffering from depression (OR=8.51, 95% CI=2.00 – 36.17, *p*=0.004) increased the risk of suicidal ideation.
